# Efficiently Degrading RhB Using Bimetallic Co_3_O_4_/ZnO Oxides: Ultra-Fast and Persistent Activation of Permonosulfate

**DOI:** 10.3390/molecules30102237

**Published:** 2025-05-21

**Authors:** Bai Sun, Rui Liu, Fengshou Zhao, Shengnan He, Yun Wang, Xiangxiang Wang, Hao Huang, Mingjian Yi, Shuguang Zhu

**Affiliations:** 1 Engineering Research Center of Building Energy Efficiency Control and Evaluation, Ministry of Education, College of Environment and Energy Engineering, Anhui Jianzhu University, Hefei 230601, China; 2 Environmental Materials and Pollution Control Laboratory, Hefei Institute of Physical Science, Chinese Academy of Sciences, Hefei 230031, China

**Keywords:** Co_3_O_4_/ZnO composite, persulfate activation, RhB degradation, bimetallic synergy

## Abstract

To address the issues of poor Co^2+^ regeneration and limited interfacial electron transfer in heterogeneous catalytic systems, this study proposes the synthesis of highly efficient and stable Co_3_O_4_/ZnO composites through the pyrolysis–oxidation reaction of Co/Zn MOFs for the degradation of rhodamine B (RhB) using activated peroxymonosulfate (PMS). The results confirmed that the catalyst exhibited a high electron transfer capacity, and the synergistic effect between the bimetals enhanced the reversible redox cycle of Co^3+^/Co^2+^. Under optimal conditions, complete removal of RhB was achieved in just 6 min using the Co_3_O_4_/ZnO composite, which demonstrated excellent stability after five cycles. Furthermore, the catalyst exhibited a high degradation efficiency in real water samples with a total organic carbon (TOC) removal rate of approximately 65% after 60 min. The electrochemical measurements, identification of active species, and X-ray photoelectron spectroscopy (XPS) analysis revealed that non-radicals (^1^O_2_ and direct charge transfer) played a major role in the degradation of RhB. Finally, the potential mechanisms and degradation pathways for RhB degradation using this catalyst were systematically investigated. This study opens new avenues for the development of efficient and stable PMS catalysts, and provides insights into the preparation of other emerging metal oxides.

## 1. Introduction

RhB, a common azo dye, poses considerable toxicity risks even at low concentrations in wastewater [1]. As the economy rapidly advances, traditional treatment methods can hardly meet discharge regulations for dye wastewater. Consequently, effectively treating RhB in dye wastewater has become a primary focus of research in the water treatment industry today. Peroxymonosulfate-based advanced oxidation processes (AOPs) are regarded as highly effective for the removal of persistent organic pollutants from water. This is due to their strong redox potential (2.5–3.1 eV), extended lifetime (30–40 s), and wide pH range (3–9) [2]. It is known that there are many methods to activate persulfate [3]. Among these, non-metallic activation is often inefficient, while thermal and photoactivation entail high costs due to the need for external energy input [4]. Alkali activation necessitates rigorous equipment and operational requirements, and strong bases can potentially pose environmental risks [5]. In contrast, transition metals, especially the transition metal ions and their oxides represented by cobalt, are widely used because of their straightforward operation and high energy efficiency.

Co_3_O_4_ serves as a heterogeneous catalyst with a remarkable ability to remove organic pollutants that are difficult to degrade [6]. However, there remains challenges, such as poor regeneration of Co^2+^ ions and limited interfacial electron transfer in heterogeneous catalytic systems. Several studies have demonstrated that bimetallic catalysts are promising solutions for the challenges associated with Co_3_O_4_ catalytic systems, owing to their combinatorial versatility and high catalytic activity. For instance, Reda et al. prepared ZnO-Co_3_O_4_ through mechanosynthesis by grinding the ZnO precursor, sodium hydroxide, and Co_3_O_4_ at room temperature, demonstrating that heterojunctions between bimetallics positively influence PMS activation [7]. Niu et al. dispersed metallic cobalt onto a non-toxic and environmentally friendly carbon-based carrier and combined it with another active metal to create Co_3_O_4_-Mn_3_O_4_ @ACFs, which exhibited highly efficient PMS utilization [8]. However, shorter mechanical treatment times may adversely affect the catalytic performance of these catalysts, and the introduction of carriers often leads to challenges, such as in situ growth and interfacial stability of the catalysts. Therefore, it is crucial to find more stable and efficient methods to prepare bimetallic catalysts in the current study.

Metal–organic frameworks (MOFs) are unique three-dimensional materials that utilize organic ligands to immobilize metal centers. They have gained an important position in the synthesis of functional materials, such as metal oxides. MOFs not only enhance the stability of the metal source center by creating a three-dimensional spatial structure but also avoid the introduction of support, thus reducing the new problems caused by interface mass transfer resistance [9]. Furthermore, the derivatives produced from the pyrolysis of MOFs are anticipated to further enhance charge transfer and adsorption capabilities, which contribute positively to the performance and stability of PMS-activated systems [10]. Wang et al. prepared Co_3_O_4_/NiCo_2_O_4_ double-shelled nanocages (DSNCs) using ZIF-67 as a precursor. This approach created a broad reaction space for the interaction between peroxydisulfate (PDS) and bisphenol A (BPA), thereby accelerating the degradation of BPA [11]. Yin et al. prepared bimetallic oxides from a novel 3D-ZIF that exhibit a high electron transfer capacity and multiple reaction sites, facilitating the efficient degradation of pollutants [12]. Previous studies have demonstrated that ZnO is an n-type semiconductor, which can facilitate electron transfer from the catalyst to PMS [6]. Additionally, ZnO possesses the capability to oxidize organic pollutants and can effectively activate persulfate by leaching Zn ions (electron donors) into the solution. Thus, zinc oxide is expected to facilitate the electronic reconstruction of Co_3_O_4_, thereby enhancing its catalytic activity. Therefore, constructing Co_3_O_4_ and ZnO bimetallic composites using MOFs as precursors may be a viable approach to enhance electron transfer and improve catalytic performance.

Based on the above discussion, a novel Co_3_O_4_/ZnO composite coral-like structure was successfully prepared using Co/Zn-MOF as the precursor. Unlike the previously reported microspherical Co_3_O_4_/ZnO catalysts, the coral-like structure partially addresses the issues associated with the relatively low specific surface area and extended mass transfer pathways caused by the dense microspherical morphology. In addition, the stability and reusability of the Co_3_O_4_/ZnO composite in aqueous solution were studied, and the adaptability of the catalyst in actual water samples was discussed. Finally, a possible mechanism for RhB degradation by the Co_3_O_4_/ZnO composite through the activation of PMS has been analyzed. Additionally, potential degradation pathways were suggested based on prior studies and the degradation intermediates identified through LC-MS. This research opens the door for the development of efficient and stable Finon-like heterogeneous catalysts, boosting their effectiveness in wastewater treatment.

## 2. Results and Discussion

### 2.1. Synthesis and Characterization of Material

**Figure 1:**
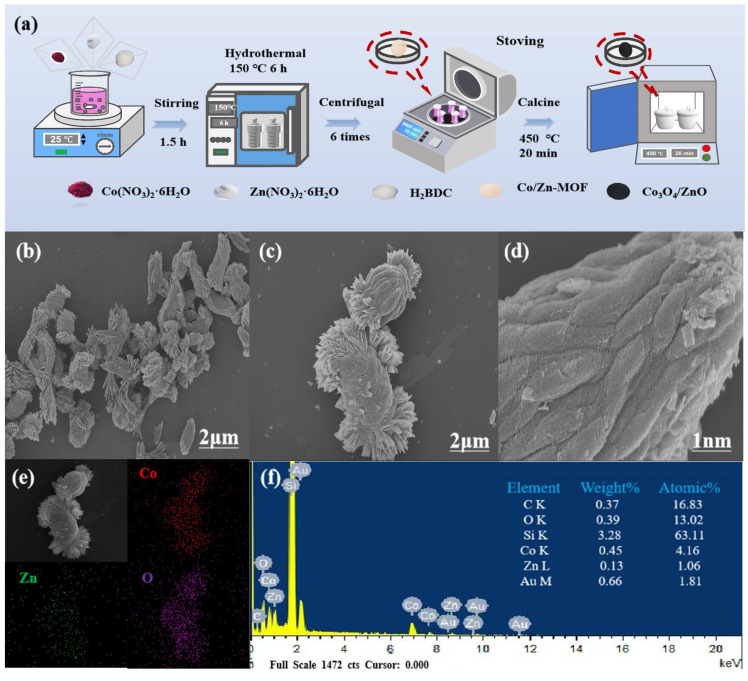
(**a**) Illustration for preparation route of Co_3_O_4_/ZnO composite. (**b**–**d**) SEM images of Co_3_O_4_/ZnO composite. (**e**) Corresponding element mappings and (**f**) energy-dispersive X-ray spectrum.

Firstly, the synthesis process of Co_3_O_4_/ZnO composites is illustrated in Figure 1a. Co(NO_3_)_2_·6H_2_O and Zn(NO_3_)_2_·6H_2_O were dissolved in a solvent mixture of ethylene glycol and DMF to create a homogeneous solution. Next, H_2_BDC was added to the solution and stirred continuously for 1 h. Subsequently, the solution was heated to 150 °C for 6 h, then cooled, washed, and dried. Finally, the precursor was calcined at 450 °C for 20 min to obtain the final product, Co_3_O_4_/ZnO. Additionally, monometallic oxides (Co_3_O_4_ and ZnO) were prepared using only one type of nitrate.

The SEM images of the single oxide are presented in Figure S1. Co_3_O_4_ exhibits a straw bale-like morphology, while ZnO displays a blade-like morphology. Notably, the Co_3_O_4_/ZnO composite reveals a coral-like morphology, and upon magnification, an aggregated blade-like morphology can be observed on the catalyst (Figure 1b–d). This observation confirms the presence of both ZnO and Co_3_O_4_ in the catalysts. Furthermore, in contrast to the previously reported microsphere-like Co_3_O_4_/ZnO catalysts, the morphology of the collapsed and porous blade-like structures is randomly stacked on the surface of the material. This configuration not only provides interconnected porous channels that reduce mass transfer resistance, but also enhances the specific surface area of the material, thereby increasing the number of active sites [6,7,13]. Moreover, the elemental distribution of Co, Zn, and O in the Co_3_O_4_/ZnO composite was revealed using EDS elemental mapping (Figure 1e). The corresponding EDS energy mapping further confirmed the inclusion of Co, Zn, and O in the Co_3_O_4_/ZnO composite (Figure 1f), which confirmed the successful preparation of the Co_3_O_4_/ZnO composite.

**Figure 2:**
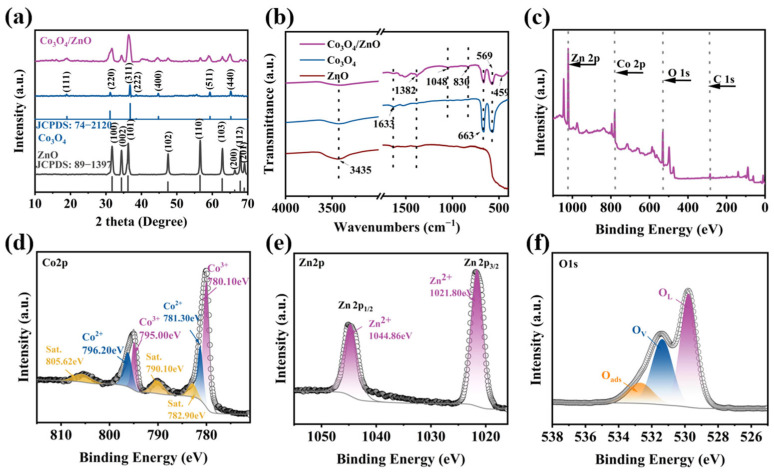
(**a**) XRD and (**b**) FTIR spectra of Co_3_O_4_, ZnO, and Co_3_O_4_/ZnO composite. (**c**) Specific XPS analysis: (**d**) Co 2p, (**e**) Zn 2p, and (**f**) O 1s.

The composition and crystal structure of the Co_3_O_4_, ZnO, and Co_3_O_4_/ZnO composites were analyzed by X-ray diffraction (XRD). As shown in Figure 2a, the characteristic diffraction peaks of both Co_3_O_4_ and ZnO were observed in the Co_3_O_4_/ZnO composites. Concretely, the diffraction peaks at 2θ = 18.9°, 31.3°, 36.8°, 38.5°, 44.8°, 59.4°, and 65.2° correspond to the (111), (220), (311), (222), (400), (511), and (440) crystalline planes of Co_3_O_4_ in the composite material, which are consistent with the JCPDS NO.74- 2120 card [14]. Additionally, the diffraction peaks at 2θ = 31.7°, 34.4°, 36.2°, 47.5°, 56.5°, 62.8°, 66.3°, 67.9°, and 69.0° correspond to the (100), (002), (101), (102), (110), (103), (200), (112), and (201) crystalline planes of ZnO, which are in tune with the ZnO previously reported (JCPDS NO. 89-1397) [15]. In addition, no characteristic peaks of significant impurities were detected, indicating that the synthesized composites were of high purity and comprised Co_3_O_4_ and ZnO.

The molecular structure and functional groups of the catalysts were analyzed using FTIR (Figure 2b). The FTIR spectra of pure Co_3_O_4_ and ZnO revealed that the peaks at 3435 and 1633 cm^−1^ correspond to the O-H stretching vibrations of water molecules [16]. Meanwhile, the peaks at 663 and 569 cm^−1^ were associated with the stretching vibrations of Co^3+^-O and Co^2+^-O in Co_3_O_4_, respectively [17]. Additionally, the broadband vibration at 459 cm^−1^ is considered the stretching vibration of Zn-O [12]. The characteristic peaks of the Co_3_O_4_/ZnO composite include 3435, 1633, 1382, 663, 569, and 459 cm^−1^, which further confirm the presence of ZnO and Co_3_O_4_ in the ZnO/Co_3_O_4_ composite. Furthermore, the weak vibration of the peak at 3435 cm^−1^ indicates that, during the high-temperature calcination process, the water molecules adsorbed on the surface were removed. No additional stretching bands were observed in the Co_3_O_4_/ZnO composite structure, indicating the high purity of the sample.

The surface chemical state of the Co_3_O_4_/ZnO composite was analyzed using X-ray photoelectron spectroscopy (XPS). The XPS survey spectrum of the Co_3_O_4_/ZnO composite indicated the presence of Co 2p, Zn 2p, C 1s, and O 1s peaks, which are consistent with the EDS results (Figure 2c). Figure 2d shows a high-resolution XPS spectrum of Co 2p, where the two main peaks at 793.5 eV and 778.6 eV correspond to Co 2p1/2 and Co 2p3/2, respectively. The peaks at 778.8 eV and 793.8 eV can be attributed to Co^3+^, while the peaks at 782.2 eV and 797.4 eV can be assigned to Co^2+^ [18]. Additionally, the peaks at 788.5 eV and 805.6 eV can be identified as satellite peaks of Co 2p, indicating the presence of Co_3_O_4_ [19]. The Zn 2p spectrum is shown in Figure 2e. The two peaks at 1044.3 and 1021.2 eV correspond to Zn 2p1/2 and Zn 2p3/2, respectively, and the spin-orbit division of Zn2p is approximately 23.0 eV. This demonstrates the form of Zn^2+^ in the Co_3_O_4_/ZnO composite, confirming the presence of ZnO in the Co_3_O_4_/ZnO composite [20]. In the XPS spectrum of O1s (Figure 2f), three independent peaks at 529.4 eV, 530.8 eV, and 532.5 eV correspond to lattice oxygen (O_L_), oxygen vacancies (O_V_), and adsorbed-state oxygen (O_ads_) on the surface, respectively [21]. These results further confirm the successful preparation of the Co_3_O_4_/ZnO composite.

**Figure 3:**
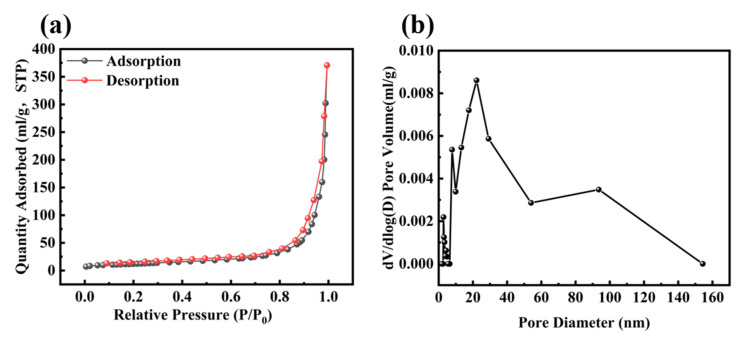
(**a**) N_2_ adsorption/desorption isotherms of Co_3_O_4_/ZnO and (**b**) pore size distribution of Co_3_O_4_/ZnO.

To study the porosity of the Co_3_O_4_/ZnO composites, the specific Brunauer–Emmett–Teller (BET) surface area and the pore size of the samples were measured, and the N_2_ adsorption desorption isotherms of the sample nanomaterials were obtained to evaluate their surface area and porous structure. The specific surface area and pore size distribution are illustrated in Figure 3a,b. The specific surface area of the Co_3_O_4_/ZnO composite was calculated using the BET equation, yielding a value of 67.87 m^2^/g. According to the IUPAC classification, Co_3_O_4_/ZnO is categorized as type IV, which indicates that it is a mesoporous nanomaterial. Furthermore, the average pore size was approximately 33–45 nm, further confirming the mesoporous nature of the catalyst.

### 2.2. Catalytic Activity of Co_3_O_4_/ZnO

Firstly, in order to exclude the possibility of direct absorption of RhB by the catalysts, the mixed solution was stirred for 20 min before adding PMS. The results showed that all catalysts had a slight absorption capacity. The catalysts effectively activated PMS when it was added to the mixed solution. More interestingly, among the various catalyst systems, the removal efficiency of RhB in the Co_3_O_4_/ZnO/PMS system was much higher than that in other catalytic systems (Figure S2a). Furthermore, the k_obs_ values for the PMS, Co_3_O_4_/ZnO composite, Co_3_O_4_/PMS, ZnO/PMS, Co_3_O_4_ + ZnO/PMS, and Co_3_O_4_/ZnO/PMS systems were 0.005, 0.006, 0.009, 0.028, 0.029, and 0.872 min^−1^, respectively (Figure S2b). The results were consistent with the above findings, demonstrating that the synergistic effect of ZnO and Co_3_O_4_ can effectively activate PMS. In addition, to further validate the excellent catalytic performance of Co_3_O_4_/ZnO composites, Table S1 presents a comparison of previously reported catalyst systems for the degradation of RhB. Clearly, the removal rate of RhB by the Co_3_O_4_/ZnO composite synthesized in this study surpassed that of most catalysts, demonstrating the superior catalytic efficacy of Co_3_O_4_/ZnO/PMS systems.

### 2.3. Effect of Different Parameters on RhB Degradation Efficiency

**Figure 4:**
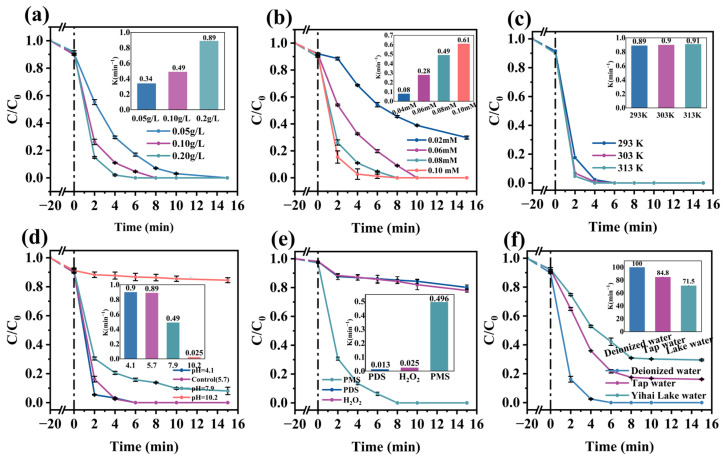
Effects of (**a**) catalyst dosage, (**b**) PMS concentration, (**c**) reaction temperature, (**d**) pH, (**e**) different oxidants catalyzed, and (**f**) different water samples on Co_3_O_4_ZnO composite to activate PMS for RhB degradation. Experimental conditions: [RhB] = 20 mg/L, [PMS] = 0.08 mM, [catalyst] = 0.1 g/L, and pH = 5.7.

Figure 4a illustrates the effect of catalyst amounts on RhB degradation. As the concentration of the Co_3_O_4_/ZnO composite increased, the RhB degradation rate showed a marked increase. At a catalyst concentration of 0.05 g/L, RhB was fully degraded within 10 min. Moreover, when the catalyst concentration was increased to 0.1 g/L, the RhB removal rate achieved 100% within 6 min. This was due to the coral-like catalyst exposing more active sites, resulting in more active species. It is important to note that the degradation rate of RhB showed a modest rise when the Co_3_O_4_/ZnO composite concentration went beyond 0.1 g/L, possibly due to saturation in the active site [22]. Therefore, considering cost-effectiveness, a catalyst dosage of 0.1 g/L can be considered optimal.

The concentration of PMS is also a crucial factor in the degradation process of RhB. Consequently, the impact of PMS concentration on the RhB degradation process was examined using a catalyst concentration of 0.1 g/L. A significant increase in degradation efficiency occurred as the PMS concentration rose from 0.04 mM to 0.08 mM (Figure 4b). Notably, the removal rate of RhB increased significantly from 61.2% to 100% at 6 min. This can be explained by the increased generation of reactive oxygen species (ROS) as the PMS concentration rose [23]. However, the elimination rate did not show a significant improvement when the PMS concentration was increased further to 0.10 mM. This may be attributed to the limited reactive sites of the catalyst [24]. Additionally, the self-scavenging of active substances (·OH and SO_4_^·−^) caused by the overuse of PMS and the production of less active radicals also obstructed the degradation of RhB [25,26]. The detailed equations are provided in Text S1.

Figure 4c depicts the effect of temperature on RhB degradation. The reaction rate of RhB degradation increases with temperature. This can be attributed to the high temperature increasing the collision frequency of the Co_3_O_4_/ZnO composite, PMS, and RhB [27]. Additionally, the activation energy (Ea) of the Co_3_O_4_/ZnO/PMS system was calculated using the Arrhenius equation. The details of the calculations are provided in Text S2. As shown in Figure S3, the activation energy for RhB degradation was 2.97 kJ/mol, which is lower compared to other MOF-derived and Co_3_O_4_-based catalysts, such as CuBTC-derived CuO, MIL-53(Fe)-derivatized CuFe_2_O_4_/Fe_2_O_3_, ZIF-67-derivatized Co_3_O_4_/NiCo_2_O_4_, carbon-loaded cobalt oxides, ZIF-67-derived magnetic carbon, and so on (Table S2). The lower Ea value for the Co_3_O_4_/ZnO composite indicates a stronger catalytic activation ability in the degradation process of RhB.

In practice, the solution pH is one of the determinants of catalytic performance. This could be due to the fact that the solution pH affects the form of PMS and the zeta potential of the Co_3_O_4_/ZnO composite, thus influencing the generation of the reactive radical species. Figure 4d illustrates the effect of the initial pH on RhB degradation. RhB is completely degraded in acidic solutions within 15 min. However, the removal of RhB was inhibited to some extent when the pH reached 10.2. This indicates that an alkaline condition is not favorable for the degradation of RhB by the Co_3_O_4_/ZnO composite. These effects could be attributed to the following points: (i) In alkaline solutions, HSO_5_^−^ is broken down into inactive substances (Equation (S8)) [28], reducing available radicals for RhB degradation. (ii) RhB is negatively charged in high pH (pH 10.2) solutions, leading to significant electrostatic repulsion between the surface of the Co_3_O_4_/ZnO composite and the RhB ions in the solutions. This interaction results in a decrease in RhB degradation. Furthermore, the zeta potential of the Co_3_O_4_/ZnO composite was measured at different pH conditions. The experimental results are shown in Text S3.

In addition, the effects of different oxidizing agents (PMS, PDS, and H_2_O_2_) on the degradation of RhB were investigated. Figure 4e clearly shows that the addition of PMS, PDS, and H_2_O_2_ to the reaction solution resulted in RhB degradation rates of 100%, 22.18%, and 20.31%, respectively. Moreover, the k_obs_ values were 0.49, 0.02, and 0.01 min^−1^, respectively. This phenomenon can be attributed to the fact that PDS with symmetry is not easily activated [29], and H_2_O_2_ can only produce a weaker radical (·OH) (Equation (S9)). Furthermore, when the concentration of H_2_O_2_ is excessively high, ·OH is readily removed (Equations (S10) and (S11)) [30].

To evaluate the practical applicability of the Co_3_O_4_/ZnO/PMS system, the effects of various inorganic anions on RhB removal efficiency (NO_3_^−^, Cl^−^, SO_4_^2−^, and HCO_3_^−^) at concentrations of 0, 10, 50, and 100 mM were investigated. As shown in Figure S5a, The presence of NO_3_^−^ had a negligible effect on RhB degradation. Cl^−^ showed a slight decrease in RhB degradation efficiency, and the degradation curves of different concentrations of Cl^−^ almost overlapped (Figure S5b). This could be attributed to Cl^−^ scavenging SO_4_^·−^ in the reaction system, resulting in the generation of active species with a lower oxidation ability (Equations (S12) and (S13)). Additionally, PMS might react with Cl^−^ to produce HOCl with a lower oxidation ability, leading to a slight decline in contaminant degradation (Equation (S14)) [31].

However, SO_4_^2−^ exhibited a slight inhibitory effect on RhB degradation experiments, and the k_obs_ values decreased from 0.89 to 0.1min^−1^ (Figure S5c). These could be attributed to its ability to scavenge active species in the reaction system. Additionally, the competition between SO_4_^2−^ and RhB for active sites also reduced the efficiency of RhB degradation [32]. Notably, HCO_3_^−^ showed significant inhibition of RhB degradation. The removal efficiency of RhB decreased notably when the HCO_3_^−^ concentration reached 10 mM, and the k_obs_ values decreased from 0.89 to 0.01min^−1^ (Figure S5d). This might be due to the fact that HCO_3_^−^ acts as a quencher of SO_4_^·−^ (Equation (S15)). However, the elimination rate slightly improved as the HCO_3_^−^ concentration increased to 50 mM and 100 mM. This phenomenon could be explained by the reaction of HCO_3_^−^ with persulfate to form HCO_4_^−^, which improves the removal efficiency of RhB [33]. Combined with the inhibitory effects of four inorganic anions on the catalytic degradation capability of Co_3_O_4_/ZnO, it is evident that the material exhibits a certain level of resistance to anionic interference.

In addition, the effect of various water samples on the degradation of RhB by the catalyst was examined to evaluate the catalyst’s suitability. Water from the lake on campus (TOC concentration of 33 mg/L) and tap water (TOC concentration of 20 mg/L) were used as water samples for the degradation experiments. The degradation rates for 20 mg/L RhB were 100% in deionized water, 84% in tap water, and 71% in campus lake water, with corresponding k_obs_ values of 0.48, 0.24, and 0.21 min^−1^, respectively (Figure 4f). As noted, various ions can reduce the efficiency of RhB degradation in the system to some extent, depending on their concentrations. Consequently, the differing types and concentrations of substances in various water samples led to the observed variations in RhB removal performance in the Co_3_O_4_/ZnO/PMS system. These findings show that, although the degradation performance of RhB in a complex water environment was somewhat disrupted, the effective removal capability was still preserved, suggesting the practical applicability of the prepared catalyst. Additionally, the total organic carbon (TOC) removal rate was monitored throughout the experiment (Figure S6). The findings indicated that the TOC removal rate reached approximately 65% within 60 min, ensuring the efficient mineralization of the pollutants and further validating the effective applicability of the Co_3_O_4_/ZnO/PMS system.

### 2.4. Reusability and Stability of Co_3_O_4_/ZnO Composite

The reusability of catalysts is one of the key factors to improve cost-effectiveness. Under identical conditions (20 mg/L RhB, 0.1 g/L Co_3_O_4_/ZnO composite, 0.08 mM PMS, and pH = 5.7), five experiments were conducted to evaluate the degradation of RhB and assess the reusability of the Co_3_O_4_/ZnO composite. As presented in Figure S7a, the RhB removal efficiency remained largely constant during the five cycle experiments. This result indicated that the Co_3_O_4_/ZnO composite had good reusability. To further demonstrate the stability of the Co_3_O_4_/ZnO composite, the catalysts before and after use were analyzed by FTIR spectroscopy and XPS (Text S5).

### 2.5. Mechanism Study

To further demonstrate the synergistic catalytic effect of Co_3_O_4_ and ZnO, CV and EIS tests were conducted on Co_3_O_4_, ZnO, and the Co_3_O_4_/ZnO composite. Firstly, in order to optimize the current profile, the effective electrochemical surface area (ECSA) was calculated by conducting cyclic voltammetry (CV) tests on Co_3_O_4_, ZnO, and the Co_3_O_4_/ZnO composites at various scanning rates ranging from 10 to 50 mV/s (Figure S8a–c) [34,35]. The flat region without obvious redox peaks (0.15–0.25 V vs. RHE) was selected to confirm that the current was linearly related to the scan rate across different scan rates (Figure S9) [36]. The double-layer capacitances (*C_dl_*) of 0.4897, 0.7526, and 0.8956 mF cm^−2^ were calculated for Co_3_O_4_, ZnO, and the Co_3_O_4_/ZnO composites, respectively, at the selected potential of 0.2 V vs. RHE [37,38]. The *ECSA* values for Co_3_O_4_, ZnO, and Co_3_O_4_/ZnO were then calculated using Equation (1), yielding results of 12.24, 18.82, and 22.39 cm^2^, respectively.

(1) $$ECSA=\frac{C_{dl}}{C_{s}}$$

where *C_dl_* is the double-layer capacitance (calculated from the slope of the CV curve). *C_s_* is the normalized capacitance value per unit area (taken as 0.04 mF cm^−2^) [39].

**Figure 5:**
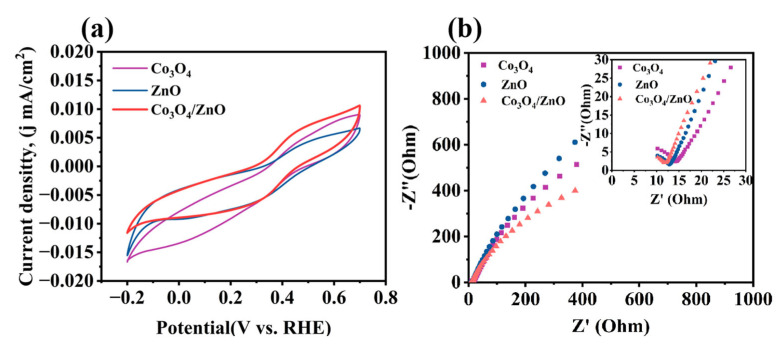
(**a**) CV curves of Co_3_O_4_, ZnO, Co_3_O_4_/ZnO composite, respectively; (**b**) EIS spectrum of Co_3_O_4_, ZnO, and Co_3_O_4_/ZnO composite, respectively.

The optimized current curves are presented in Figure 5a, and the CV curves of ZnO exhibit distinct redox peaks. However, the redox peaks of Co_3_O_4_ are less pronounced, with the oxidation and reduction peaks being the narrowest, indicating poorer electron transfer cyclability. Notably, the oxidation and reduction peaks of Co_3_O_4_/ZnO composites are significantly more pronounced and exhibit larger peak areas compared to those of ZnO and Co_3_O_4_. This indicates that the Co_3_O_4_/ZnO composites exhibit improved electron transfer recoverability, which facilitates electron transfer and the generation of active species. Meanwhile, in comparison to Co_3_O_4_, the arc radius of the Co_3_O_4_/ZnO composite was smaller (Figure 5b). This shows that the Co_3_O_4_/ZnO composite exhibits reduced charge transfer resistance (R_ct_), resulting in a faster electron transport rate. This enhanced electron transport rate is beneficial for facilitating PMS activation and improving pollutant degradation [23].

**Figure 6:**
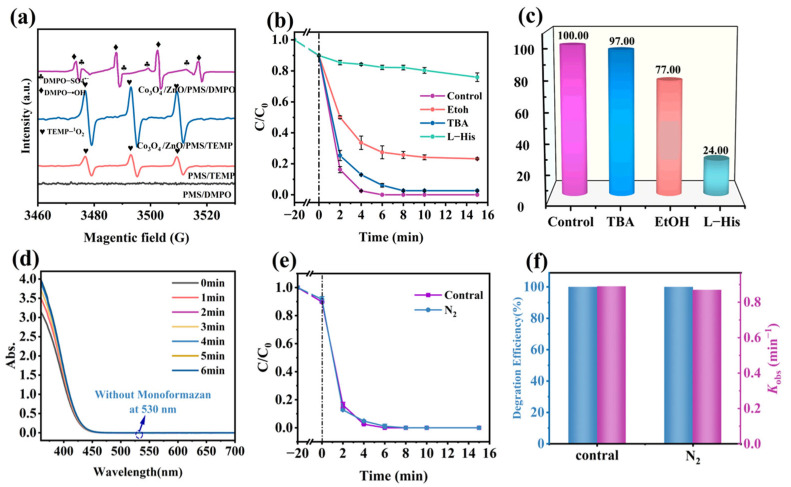
(**a**) EPR spectra of DMPO-·OH, DMPO- SO_4_^·−^, and TEMP-^1^O_2_ in Co_3_O_4_/ZnO/PMS system. (**b**) UV-Visible absorption spectra of the variation of monoformazan. (**c**,**d**) Removal efficiency of RhB with the addition of different scavengers in Co_3_O_4_/ZnO/PMS system. (**e**,**f**) Effect of dissolved oxygen on degradation of RhB in Co_3_O_4_/ZnO/PMS system. Reaction conditions: [RhB] = 20 mg/L, [PMS] = 0.08 mM, [catalyst] = 0.1 g/L, [DMPO] = 5 mM, [EtOH] = [TBA] = [L-his] = 80 mM, and pH = 5.7.

To detect the active species present in the Co_3_O_4_/ZnO/PMS system, EPR experiments and radical quenching tests were conducted. In the EPR experiments, DMPO and TEMP were employed as scavengers for SO_4_^·−^, ·OH, ·O_2_^−^, and ^1^O_2_, respectively [40]. With the findings shown in Figure 6a, the PMS/DMPO system did not display any radical signal, while the PMS/TEMP system exhibited weak characteristic peaks with a 1:1:1 intensity ratio, which can be attributed to the self-decomposition capability of PMS [41]. It is noteworthy that the characteristic peaks with a 1:1:1 intensity ratio were obviously observed when both the Co_3_O_4_/ZnO composite and PMS were introduced into the reaction solution, which is a signature of ^1^O_2_. Additionally, the characteristic peaks with an intensity ratio of 1:2:2:1 were observed in the Co_3_O_4_/ZnO/PMS/DMPO system, indicating the presence of ·OH. Meanwhile, the weak signals around these peaks were the typical peaks of DMPO-SO_4_^·−^ [3]. Subsequently, ·O_2_^−^ was further identified in the Co_3_O_4_/ZnO/PMS system using NBT as a molecular probe. A concentration of 40 μM NBT was introduced to the solution, and samples were collected every minute. The UV-Visible absorption spectra indicated that there was no response peak at 530 nm during the reaction (Figure 6b), further confirming that no ·O_2_^−^ was generated in the system. Therefore, SO_4_^·−^, ·OH, and ^1^O_2_ were produced in the Co_3_O_4_/ZnO/PMS system, and no ·O_2_^−^ was formed.

In addition, in order to further determine the major active species, radical quenching tests were conducted (Figure 6c). In these tests, EtOH was utilized as a scavenger for ·OH and SO_4_^·−^, while TBA served as a typical scavenger for ·OH. L-histidine was employed as a selective scavenger to capture ^1^O_2_ [12]. With the findings shown in Figure 6d, the degradation efficiency of RhB was inhibited to varying extents with the addition of different quenchers. Specifically, the presence of TBA reduced the RhB removal rate from 100% to 97%, indicating that SO_4_^·−^ was not the predominant active species. In contrast, the introduction of ethanol decreased the RhB removal rate from 100% to 77%, indicating the significant role of ·OH in the degradation process. Notably, L-histidine substantially impeded the removal of RhB, with the rate dropping from 100% to 24%, highlighting ^1^O_2_ as the primary active species.

Previous studies have shown that ·O_2_^−^ is a significant intermediate in the production of ^1^O_2_ [42]. However, the NBT and EPR experiments indicated that ·O_2_^−^ is not present. Consequently, ^1^O_2_ is not derived from ·O_2_^−^. Figure 6a illustrated that the TEMP-^1^O_2_ peak signal can be detected using only PMS, suggesting that a portion of the ^1^O_2_ is generated from the self-decomposition of PMS. In addition, to investigate whether ^1^O_2_ originates from oxygen that is dissolved or produced during the reaction process, N_2_ was continuously injected into the Co_3_O_4_/ZnO/PMS system. The findings shown in Figure 6e indicate that the RhB degradation curves of the system with N_2_ nearly overlapped with those of the system without N_2_, and both the degradation rate and the k_obs_ showed little to no change (Figure 6f). This suggests that the contribution of dissolved or generated oxygen to the production of ^1^O_2_ during the reaction is negligible. Therefore, ^1^O_2_ is self-composed from PMS.

**Figure 7:**
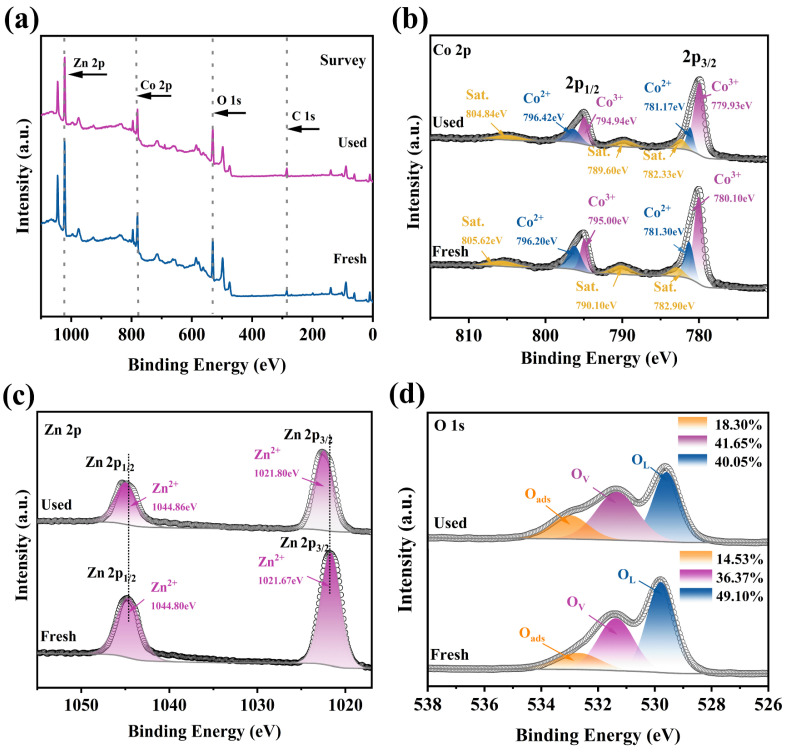
(**a**) Survey spectrum, (**b**) Co 2p, (**c**) Zn 2p, and (**d**) O 1s in XPS spectra of Co_3_O_4_/ZnO composite catalyst before and after reaction (Reaction conditions: [RhB] = 20 mg/L, [PMS] = 0.08 mM, [catalyst] = 0.1 g/L, [DMPO] = 5 mM, and pH = 5.7).

In order to investigate how the Co_3_O_4_/ZnO complex activates PMS, the valence changes of both fresh and used samples were analyzed using XPS. As seen in the high-resolution XPS spectrum of Co 2p before and after the reaction (Figure 7b), the proportion of Co(II) reduced from 33.19% to 25.47%, while Co(III) increased from 66.81% to 74.53%. This suggests that Co(II) might convert to Co(III) after participating in the activation of PMS. Figure 7c displays the high-resolution XPS spectrum of Zn 2p before and after the reaction. Clearly, the Zn(II)-related peaks completely occupied the d orbitals. This indicates that ZnO could serve as an active electron helper to provide more electrons to the active site, thus accelerating the electron transfer process during the reaction. Furthermore, compared with pure ZnO (Zn 2p1/2 at 1044.89 eV and Zn 2p3/2 at 1021.8 eV), the Zn 2p peaks of the Co_3_O_4_/ZnO composite shifted towards a lower energy [43]. This further indicated an electron interaction between ZnO and Co_3_O_4_ [7]. Also noteworthy, the main peaks of Zn 2p shifted towards a higher energy after the reaction, suggesting the partial involvement of Zn(II) in the degradation process [44].

Figure 7d analyzed the high-resolution XPS spectrum of O 1s before and after the reaction. The results showed that the O_ads_ on the surface of Co_3_O_4_/ZnO composite increased from 14.53% to 18.30% after the reaction, indicating that the hydroxylation reaction occurred on its surface, which resulted in more effective active sites and facilitated the activation of PMS [45]. Additionally, the O_L_ content of the Co_3_O_4_/ZnO composite decreased from 49.10% to 40.05%, while the O_V_ content increased from 36.37% to 41.65% after the reaction, indicating that the oxygen atoms in the Co_3_O_4_/ZnO composite participate in PMS activation [46].

**Figure 8:**
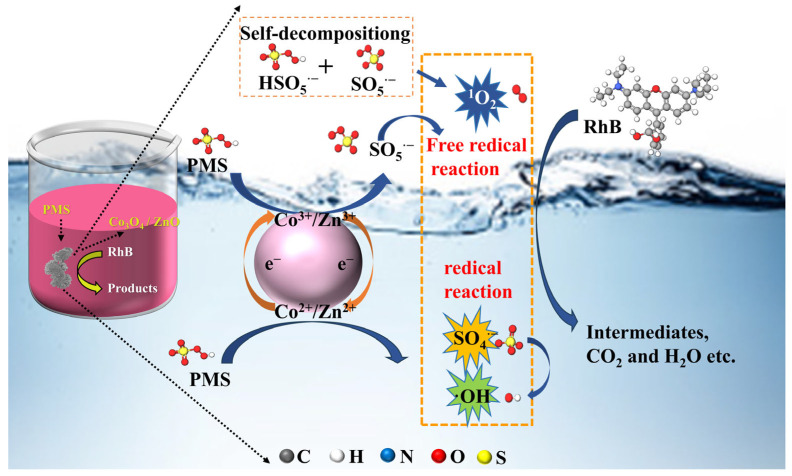
Illustration of possible mechanism of pollutant degradation in Co_3_O_4_/ZnO/PMS reaction process.

Based on the results of the electrochemical measurements, XPS analysis, and identification of active species, it is proposed that the mechanism for the degradation of RhB by the Co_3_O_4_/ZnO/PMS catalytic system primarily involves two pathways (Figure 8). In the free radical oxidation pathway, Co^2+^ and Zn^2+^ serve as electron donors, reacting directly with PMS to generate SO_4_^·−^ or ·OH (Equations (S16) and (S17)). Most of the SO_4_^·−^ are converted to ·OH, and only a small fraction of SO_4_^·−^ is directly involved in the degradation of RhB (Equations (S18) and (S19)). In the non-radical process, Co^3+^ and Zn^3+^ serve as electron acceptors, facilitating the decomposition of PMS to SO_5_^·−^ while simultaneously regenerating Co^2+^ and Zn^2+^. Subsequently, SO_5_^·−^ reacts further with water or PMS to promote the formation of ^1^O_2_ (Equations (S20)–(S23)). In addition, ^1^O_2_ can also be generated by the self-decomposition of PMS and the reaction of lattice oxygen with PMS (Equations (S24) and (S25)). The formation of a heterojunction between Co_3_O_4_ and ZnO accelerated the electron transfer process during the reaction, further enhancing the regeneration of Co^2+^ (Equation (S26)). Ultimately, RhB that was enriched on the surface of the catalyst was degraded to CO_2_ and H_2_O by radicals (SO_4_^·−^,·OH) and nonradicals (^1^O_2_ and direct charge transfer) generated in the Co_3_O_4_/ZnO/PMS system (Equation (S27)). The equations of the mechanism are detailed in Text S6. In addition, Table S4 compares the free radical and non-free radical pathways.

### 2.6. Possible Degradation Intermediates and Pathways

Firstly, the UV-Vis spectral changes observed during the oxidation of RhB in the Co_3_O_4_/ZnO/PMS system were analyzed (Figure S10). The absorbance of RhB at 554 nm gradually decreased and nearly vanished after 5 min of reaction. Meanwhile, the color of the reaction solution transitioned from its initial pink hue to nearly colorless. In addition, the maximum absorption peak hardly shifted to blue during this process. Therefore, disruption of the RhB conjugate structure is regarded as the primary mechanism for its decolorization.

**Figure 9:**
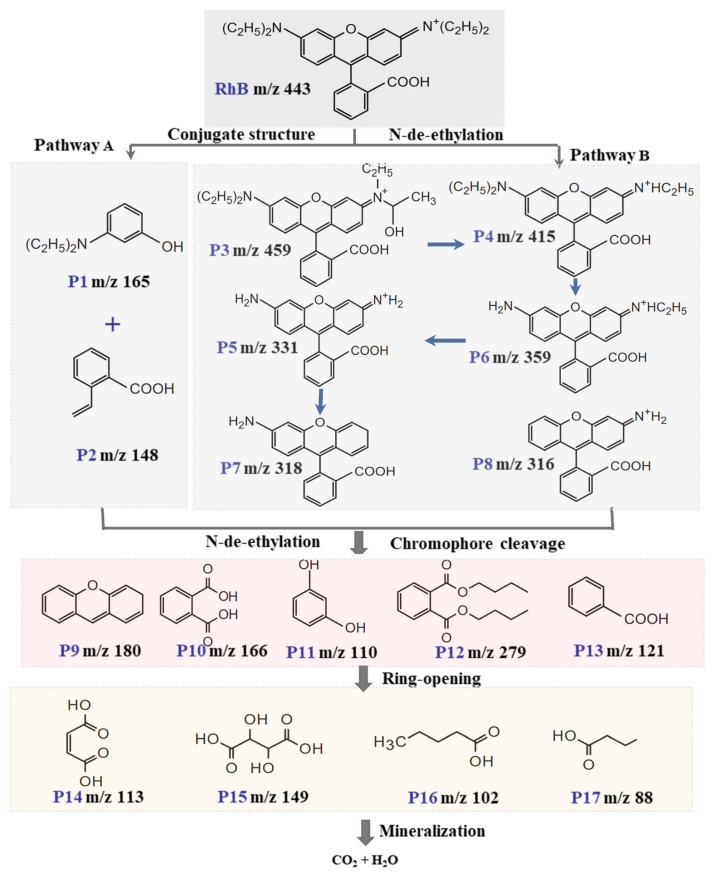
Possible degradation pathways of RhB in Co_3_O_4_/ZnO/PMS system.

LC-MS analysis was used to examine the potential intermediates of RhB in the Co_3_O_4_/ZnO/PMS system over various reaction times (Figure S11). The findings showed that the peak intensity of RhB (*m*/*z* = 443) gradually decreased as the reaction progressed, ultimately disappearing completely after 6 min, which suggests that the RhB structure was entirely destroyed. Seventeen potential intermediates were identified in the Co_3_O_4_/ZnO/PMS system, and their structural formulas are presented in Table S3. The LC-MS analysis and previous studies indicate that RhB degradation primarily occurs through four processes: n-desethylation, structural cleavage of the chromophore, ring opening, and mineralization [47,48]. However, n-desethylation and chromophore cleavage typically occur as competing processes [49]. Two degradation pathways are possible in the Co_3_O_4_/ZnO/PMS system, as illustrated in Figure 9. In pathway A, ROS preferentially attack the conjugated structure of RhB, leading to the formation of P1 (*m*/*z* = 165, C_10_H_15_NO) and P2 (*m*/*z* = 148, C_9_H_8_O_2_), which leads to rapid decolorization. These are further degraded through n-de-ethylation and ring-opening processes, producing a series of small-molecule organic acids, including P9 (*m*/*z* = 180, C_13_H_10_O), P10 (*m*/*z* = 166, C_8_H_6_O_4_), P11 (*m*/*z* = 110, C_6_H_6_O_2_), P14 (*m*/*z* = 113, C_4_H_4_O_4_), and P15 (*m*/*z* = 149, C_4_H_6_O_6_). These compounds are eventually mineralized into harmless CO_2_, water, etc. In the B pathway, RhB is first hydroxylated to yield the intermediate P3 (*m*/*z* = 459, C_28_H_31_N_2_O_4_). Subsequently, the ethanol group is removed to produce P4 (*m*/*z* = 415, C_26_H_27_O_3_N_2_^+^). Further elimination of the ethyl and amino groups results in the formation of P5 (*m*/*z* = 359, C_22_H_19_O_3_N_2_^+^), P6 (*m*/*z* = 331, C_20_H_15_O_3_N_2_^+^), P7 (*m*/*z* = 318, C_20_H_16_NO_3_), and P8 (*m*/*z* = 316, C_20_H_16_O_3_N^+^). The organic contaminants are then further degraded, and the chromophores are cleaved to form P12 (*m*/*z* = 279, C_16_H_22_O_4_) and P13 (*m*/*z* = 121, C_7_H_6_O_2_), leading to the decolorization of RhB. Subsequent ring-opening reactions produce small molecules, such as P16 (*m*/*z* = 102, C_5_H_10_O_2_) and P17 (*m*/*z* = 88, C_4_H_8_O_2_) intermediates. Ultimately, these small molecules can be mineralized into carbon dioxide, water, and other byproducts.

## 3. Materials and Methods

### 3.1. Materials

Terephthalic acid (H_2_BDC) and nitrotetrazolium blue chloride (NBT) were obtained from Aladdin Co., China. Permonosulfate (PMS) and perdisulfate (PDS) were supplied by Shanghai McLean Biochemical Technology Co., Ltd., (Shanghai, China). Cobalt nitrate hexahydrate (Co(NO_3_)_2_·6H_2_O), zinc nitrate hexahydrate (Zn(NO_3_)_2_·6H_2_O), N,N-dimethylformamide (DMF), ethylene glycol (CH_2_OH), hydrogen peroxide (H_2_O_2_), Rhodamine B (C_28_H_31_ClN_2_O_3_), sodium thiosulfate (Na_2_S_2_O_3_), histidine (C_6_H_9_N_3_O_2_), anhydrous ethanol (C_2_H_6_O), tert-butanol (TBA), sodium hydroxide (NaOH), potassium ferricyanide (K_3_[Fe(CN)_6_]), and potassium chloride (KCl) were purchased from Sinopharm Chemical Reagent Co., Ltd., (Shanghai, China). Ultrapure water (18.2 MΩ cm) was used for all the experiments. All reagents were of analytical grade and were used without further processing.

### 3.2. Synthesis of Co_3_O_4_/ZnO Composite

Firstly, 0.25 g of Co(NO_3_)_2_·6H_2_O and 0.25 g of Zn(NO_3_)_2_·6H_2_O were dissolved in 65 mL of a mixed solvent (DMF/ethylene glycol = 8/5). The mixture was agitated for 30 min, resulting in a clear solution. Then, 0.15 g of H_2_BDC was introduced into the mixed metal salt solution and continuously stirred for 1 h to ensure uniformity. Subsequently, the homogenized solution was transferred to a 100 mL polytetraffuoroethylene autoclave and heated at 150 °C for 6 h. The resulting precipitate was rinsed three times each with DMF and ethanol, followed by drying in an oven for 12 h. The sample was named Co/Zn-MOF. Finally, to convert the Co/Zn-MOF precursors into coral-like Co_3_O_4_/ZnO composite derivatives, they were subjected to calcination at 450 °C for 20 min. Under the same experimental conditions, the monometallic oxides (Co_3_O_4_ and ZnO) were prepared with only one nitrate (0.25 g). Figure 1a illustrates the schematic of the Co_3_O_4_/ZnO composite synthesis procedure.

### 3.3. Experiments on the Catalytic Activity of RhB

For the purpose of analyzing the catalytic behavior of Co_3_O_4_/ZnO composite, degradation experiments were conducted using RhB as the pollutant. Firstly, 2 mg of catalyst (100 mg/L) was mixed with 20 mL of RhB solution (20 mg/L). The homogenized solution was obtained by ultrasonic treatment for 5 min. Then, the homogeneous solution was transferred to the agitator for 15 min to reach the absorption–desorption equilibrium. Then, 40 μL of PMS solution (25 g/L) was introduced into the mixture. The pH at the start of the experiment was controlled by the addition of 0.1 M NaOH and 0.1 M HCl. At the same time intervals, the sample was collected and added with a precise amount of Na_2_S_2_O_3_ immediately to quench the degradation reaction, and then filtered using a 0.22 μm needle filter before analyzing the catalytic activity. The concentration of RhB was quantified at 554 nm with a UV-Vis spectrophotometer. The degradation rate (η) of RhB and the pseudo-first-order reaction kinetics were calculated as follows:

(2) $$\eta \left(\%\right)=\frac{\left(C_{0}-C_{t}\right)}{C_{t}}\times 100\%$$

(3) $$-ln(\frac{C_{t}}{C_{0}})=k\times t$$

where C_0_ (mg/L) and C_t_ (mg/L) represent the concentration of RhB at the beginning and at a certain time, respectively. k (min^−1^) represents the pseudo-first-order reaction rate constant.

In addition, the effects of catalyst dosage, PMS concentration, pH, temperature, and inorganic anions on RhB degradation were explored. The catalyst’s reusability was assessed through five cycles of degradation tests. All tests were carried out in duplicate and repeated a minimum of two times.

### 3.4. Characterization of Composites

The morphologies of the specimens were characterized by scanning electron microscopy (SEM, AURIGA, ZEISS Company, Oberkochen, Germany) coupled with energy-dispersive spectrometer (EDS, INCA X-Max 50, ZEISS Company, Germany). X-ray diffraction (XRD, PANalytical, Almelo, The Netherlands) was used to analyze the structural features of the catalysts. The chemical composition and the state of the catalysts were investigated using X-ray photoelectron spectroscopy (XPS, Thermo, Waltham, MA, USA). The functional groups of the catalysts were revealed using Fourier transform infrared spectroscopy (FTIR, Nexus-870, Thermo Nicolet, Waltham, MA, USA).

### 3.5. Analytical Methods

The pH was monitored using a pH meter (PHS-3C, Yidian, Shanghai, China). The reactive oxygen species in the Co_3_O_4_/ZnO/PMS system were identified through electron paramagnetic resonance (EPR) spectroscopy, employing DMPO and TEMP as free radical scavengers. To further assess the contribution of these reactive species to RhB degradation, EtOH, TBA, and L-histidine were utilized to quench hydroxyl radicals (·OH)/sulfate radicals (SO_4_^.−^), ·OH, and singlet oxygen (^1^O_2_), respectively. The production of superoxide anions (O_2_^.−^) during the reaction was excluded based on nitroblue tetrazolium (NBT) quantification experiments. Total organic carbon (TOC) was measured using a TOC analyzer (TOC-L CPH, Shimadzu, Japan). The intermediates produced by RhB degradation were identified using liquid chromatography-mass spectrometry (LC-MS, Agilent 1290, Agilent Technologies, Santa Clara, CA, USA). The mobile phases consisted of methanol (45%) and 0.1% formic acid (55%), with a flow rate of 0.4 mL/min and a scanning range of 80–800 *m*/*z*. CV and EIS tests were performed over a potential range of −2.0 to 0.7 V using a 0.1 M KCl solution containing 5 mM K_3_[Fe(CN)_6_] as the electrolyte, with silver chloride serving as the counter electrode and platinum wire as the reference electrode.

## 4. Conclusions

In summary, the coral-like structures of Co_3_O_4_/ZnO composite have been successfully fabricated through an easy approach. The complete removal of RhB was achieved in just 6 min using the Co_3_O_4_/ZnO composite under the optimized process conditions (0.1 g/L Co_3_O_4_/ZnO composite, 0.08 mM PMS, and pH = 5.7), and its catalytic performance significantly surpassed that of Co_3_O_4_ and ZnO. The CV and EIS tests indicate that the Co_3_O_4_/ZnO composites exhibit improved electron transfer recoverability and a faster electron transport rate. This facilitated PMS activation and improved pollutant degradation. Moreover, the Co_3_O_4_/ZnO composite exhibited good stability and reusability over five cycles of experiments. In addition, the high degradation efficiency was still retained in real water samples containing complex components, and the removal efficiencies of total organic carbon (TOC) were approximately 65% after 60 min. The EPR and active radical scavenging experiments revealed that ·OH, SO_4_^·−^, and ^1^O_2_ were involved in the degradation reaction, with ^1^O_2_ being the primary active species for RhB degradation. Finally, based on the XPS analysis of the Co_3_O_4_/ZnO composite before and after the reaction, combined with the identification of the active species, the reaction mechanisms for RhB degradation in the Co_3_O_4_/ZnO/PMS catalytic system were suggested. Overall, non-radicals (^1^O_2_ and direct charge transfer) play a key role in RhB degradation. The electronic interaction between ZnO and Co_3_O_4_ in the Co_3_O_4_/ZnO composite enhanced the electron transfer capability between the catalyst and PMS. This study provides an idea for tackling the challenges of poor Co^2+^ regeneration and limited interfacial electron transfer in heterogeneous catalytic systems.

## Data Availability

The data are contained within the article.

## Supplementary Materials

The following supporting information can be downloaded at https://www.mdpi.com/article/10.3390/molecules30102237/s1, Figure S1: SEM images of (a,b) Co_3_O_4_ and (c,d) ZnO; Figure S2: (a) RhB degradation under different experimental conditions, (b) kinetic constants of degradation in different systems; Figure S3: The Ea of Co_3_O_4_/ZnO; Figure S4: The zeta potential of Co_3_O_4_/ZnO; Figure S5: The effects of different anions on the catalytic degradation of RhB with Co_3_O_4_/ZnO: (a) Cl^−^, (b) HCO_3_^−^, (c) NO_3_^−^, and (d) SO_4_^2−^; Figure S6: The TOC removal rate of RhB in different water samples; Figure S7: (a) Stability and reusability of Co_3_O_4_/ZnO catalyst to activate PMS for RhB degradation. (b) FTIR spectra of Co_3_O_4_/ZnO before and after stability test; Figure S8: Cyclic voltammetry curves of (a) Co_3_O_4_, (b) ZnO, and (c) Co_3_O_4_/ZnO composite at different scan rates; Figure S9. Plot of scan rate versus current density variation at (a–c) 0.15 V, (d–f) 0.20 V, and (g–i) 0.25 V; Figure S10: UV–vis absorption spectra at different times during reaction; Figure S11: LC-MS chromatograms of RhB degradation intermediates at different time intervals in Co_3_O_4_/ZnO/PMS system; Table S1: Comparison of operating factors and performance of different catalyst/PMS systems for RhB degradation; Table S2: Comparison of different catalyst activation energies of organic pollutants; Table S3: Intermediates from degradation of RhB in Co_3_O_4_/ZnO/PMS system; Table S4: Comparison of free radical and non-free radical pathways; Text S1: The equation for the self-scavenging of reactive substances and the generation of less active free radicals; Text S2: The formula for Ea; Text S3: Measurements of zeta potential of Co_3_O_4_/ZnO composites at different pH values; Text S4: Equations for the effect of alkaline conditions, different oxidizing agents, and different anions on the degradation of RhB; Text S5: Comparison of FTIR spectra and xps before and after reuse; Text S6: The equations of the mechanism. References [7,11,19,22,33,45,50,51,52,53,54,55,56,57,58,59,60,61,62,63] are cited in the Supplementary Materials.
