# Supplementary material for: Efficiently Degrading RhB Using Bimetallic Co3O4/ZnO Oxides: Ultra-Fast and Persistent Activation of Permonosulfate
Source: Molecules. 2025 May 21;30(10):2237. doi: 10.3390/molecules30102237 (PMC12114238; doi:10.3390/molecules30102237)
Supplement: Supplementary file 1 [file molecules-30-02237-s001.zip › molecules-3566571-supplementary.pdf]

Supplementary Materials for

Efficiently Degrading RhB Using Bimetallic Co_3O_4/ZnO Oxides: Ultra-Fast and Persistent Activation of Permonosulfate

Bai Sun [1,2], Rui Liu [1], Fengshou Zhao [1], Shengnan He [1,*], Yun Wang [1], Xiangxiang Wang [1], Hao Huang [1,*], Mingjian Yi [1] and Shuguang Zhu [1]

[1] Engineering Research Center of Building Energy Efficiency Control and Evaluation, Ministry of Education, College of Environment and Energy Engineering, Anhui Jianzhu University, Hefei 230601, China

[2] Environmental Materials and Pollution Control Laboratory, Hefei Institute of Physical Science, Chinese Academy of Sciences, Hefei 230031, China

* Correspondence: heshengnan@ahu.edu.cn (S.H.); sgc006357@alumni.sjtu.edu.cn (H.H.)

Number of pages: 19 (including cover page)

Number of texts: 6

Number of figures: 11

Number of tables: 4

References: 20

Text S1. The equation for the self-scavenging of reactive substances and the generation of less active free radicals.

$$HSO_5^- + SO_4^{\cdot-} \rightarrow SO_4^{2-} + SO_5^{\cdot-} + H^+ \quad (S1)$$

$$HSO_5^- + \cdot OH \rightarrow SO_5^{\cdot-} + H_2O \quad (S2)$$

$$HSO_5^- + \cdot OH \rightarrow HO_2\cdot + SO_4^{2-} + H^+ \quad (S3)$$

$$HSO_5^- + SO_4^{\cdot-} + H_2O \rightarrow 2SO_4^{2-} + HO_2\cdot + H^+ \quad (S4)$$

$$SO_4^{\cdot-} + SO_4^{\cdot-} \rightarrow S_2O_8^{2-} \quad (S5)$$

$$SO_4^{\cdot-} + \cdot OH \rightarrow HSO_5^- \quad (S6)$$

Text S2. The formula for Ea

$$\ln K_{abs} = \ln A - \frac{E_a}{RT} \quad (S7)$$

where K_{obs} is the reaction rate constant at temperature T; T is the solution reaction temperature in Kelvin (K); A is the pre-exponential factor; R is the molar gas constant; and Ea is the apparent activation energy.

Text S3. Measurements of zeta potential of Co_3O_4/ZnO composites at different pH values.

The zeta potential of Co_3O_4/ZnO composite was measured at different pH conditions. Figure S4 showed that the pH_{pzc} of Co_3O_4/ZnO composite was 3.6, indicating that in pH > 3.6, the surface of Co_3O_4/ZnO composite was negatively charged. In pH 4.1 and pH 5.7 solutions, the efficiency of RhB degradation was minimally affected by the interaction between RhB molecules and the surface of Co_3O_4/ZnO composite. However, in pH 10.2 solutions, a significant negative charge was present on the surface of Co_3O_4/ZnO composite, hindering its contact with RhB[1]. Overall,

Co_3O_4/ZnO composite demonstrated excellent degradation performance in both acidic and neutral environments.

Text S4. Equations for the effect of alkaline conditions, different oxidizing agents and different anions on the degradation of RhB.

$$2HSO_5^- + 2OH^- \rightarrow 2H_2O + 2SO_4^{2-} + O_2 \quad (S8)$$

$$H_2O_2 + M(\text{II}) \rightarrow OH^- + \cdot OH + M(\text{III}) \quad (S9)$$

$$\cdot OH + H_2O_2 \rightarrow H_2O + HO_2 \cdot \quad (S10)$$

$$\cdot OH + HO_2 \cdot \rightarrow H_2O + O_2 \quad (S11)$$

$$SO_4^{\cdot -} + Cl^- \rightarrow SO_4^{2-} + Cl \cdot \quad (S12)$$

$$Cl \cdot + H_2O \rightarrow ClOH^{\cdot -} + H^+ \quad (S13)$$

$$HSO_5^- + Cl^- \rightarrow SO_4^{2-} + HOCl \quad (S14)$$

$$SO_4^{\cdot -} + HCO_3^- \rightarrow SO_4^{2-} + HCO_3 \cdot \quad (S15)$$

Text S5. Comparison of FTIR spectra and xps before and after reuse.

All characteristic functional groups remained after five cycles of RhB treatment (Figure 4b). The peaks at 3420 and 1626 cm^{-1} became slightly stronger. This can be attributed to the surface hydroxylation during the activation process, which makes the O-H signal of the catalyst stronger [2]. Additionally, a new peak at 1555 cm^{-1}, likely corresponding S-O stretching of SO_4^{2-} produced by PMS decomposition [3]. The above results indicated that the catalyst structure was largely unaffected after degradation. Furthermore, signals such as Co 2p, Zn 2p, O1s, and C1s were all found in Co_3O_4/ZnO composite before and after use, and the spectral position showed no significant shift (Figure 6a) [4], further validating the stability of Co_3O_4/ZnO composite.

Text S6. The equations of the mechanism.

$$Co^{2+}/Zn^{2+} + HSO_5^- \rightarrow Co^{3+}/Zn^{3+} + SO_4^{\cdot-} + OH^- \quad (S16)$$

$$Co^{2+}/Zn^{2+} + HSO_5^- \rightarrow Co^{3+}/Zn^{3+} + SO_4^{2-} + \cdot OH \quad (S17)$$

$$SO_4^{\cdot-} + OH^- \rightarrow SO_4^{2-} + \cdot OH \quad (S18)$$

$$SO_4^{\cdot-} + H_2O \rightarrow HSO_4^- + \cdot OH \quad (S19)$$

$$Co^{3+}/Zn^{3+} + HSO_5^- \rightarrow Co^{2+}/Zn^{2+} + SO_5^{\cdot-} + H^+ \quad (S20)$$

$$2SO_5^{\cdot-} + H_2O \rightarrow 2HSO_4^- + 1.5\,{}^1O_2 \quad (S21)$$

$$2SO_5^{\cdot-} \rightarrow S_2O_8^{2-} + {}^1O_2 \quad (S22)$$

$$2SO_5^{\cdot-} \rightarrow 2SO_4^{2-} + {}^1O_2 \quad (S23)$$

$$HSO_5^- + SO_5^{2-} \rightarrow SO_4^{2-} + HSO_4^- + {}^1O_2 \quad (S24)$$

$$Olattice + HSO_5^- \rightarrow HSO_4^- + {}^1O_2 \quad (S25)$$

$$Co^{3+} + e^- \rightarrow Co^{2+} \quad (S26)$$

$$SO_4^{\cdot-}/\cdot OH / {}^1O_2 + RhB \rightarrow intermediates \rightarrow CO_2 + H_2O \quad (S27)$$

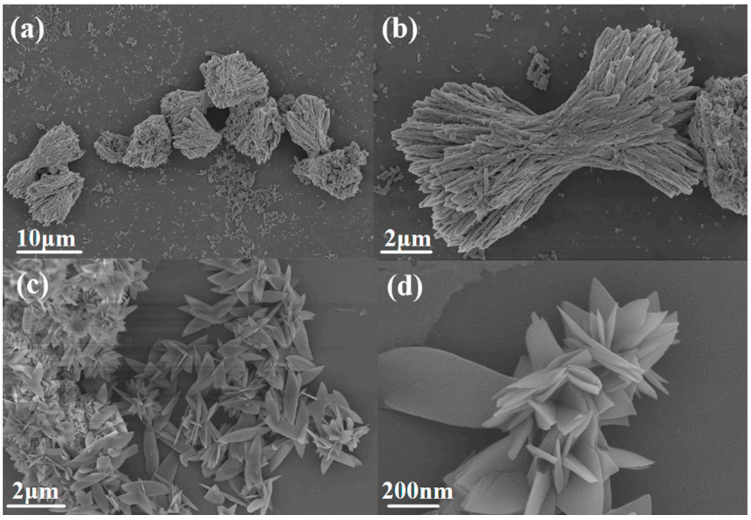

Figure S1. SEM images of (a-b) Co_3O_4 and (c-d) ZnO.

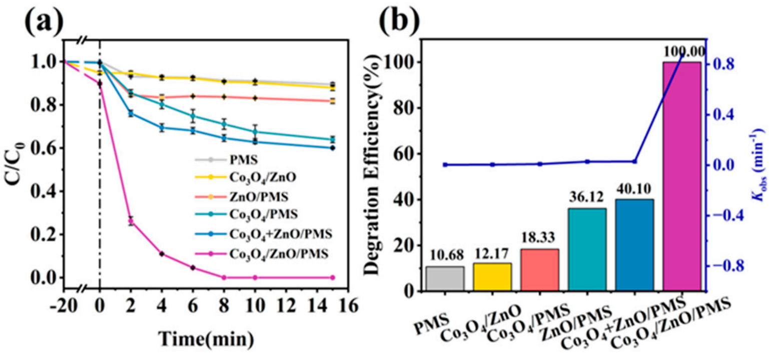

Figure S2. (a) RhB degradation under different experimental conditions, (b) kinetic constants of degradation in different systems. ([RhB] = 20 mg/L, [PMS] = 0.08 mM, [catalyst] = 0.1 g/L).

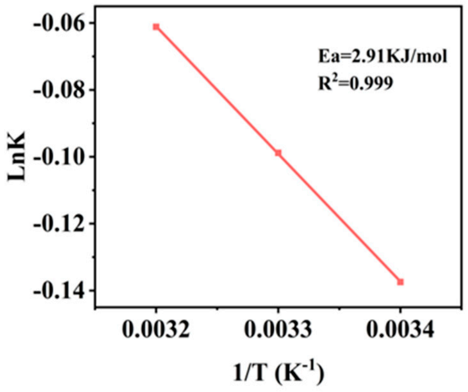

Figure S3. The Ea of Co_3O_4/ZnO.

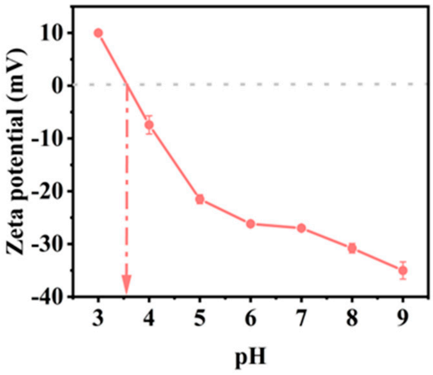

Figure S4. The zeta potential of Co_3O_4/ZnO.

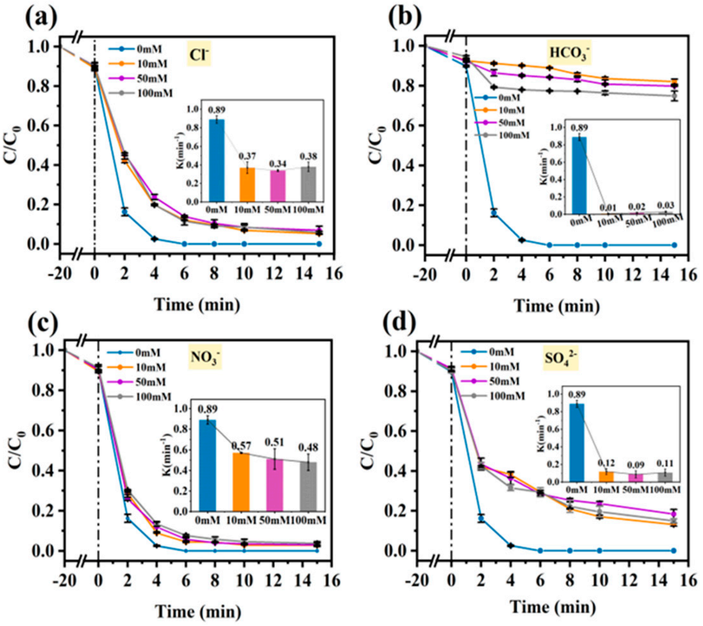

Figure S5. The effects of different anions on the catalytic degradation of RhB with Co_3O_4/ZnO: (a) Cl^-, (b) HCO_3^-, (c) NO_3^-, (d) SO_4^{2-} ([RhB] = 20 mg/L, [PMS] = 0.08 mM, [catalyst] = 0.1 g/L, pH=5.7).

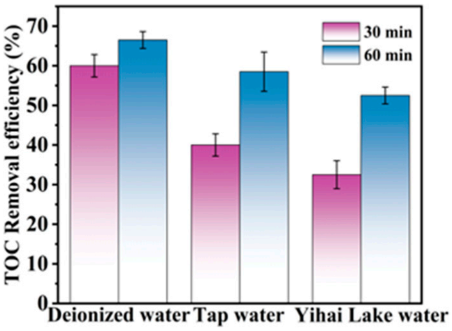

Figure S6. The TOC removal rate of RhB in different water samples. (RhB = 20 mg / L, catalyst = 0.1 g / L, PMS = 0.08 mM, T = 293K, pH = 5.7) .

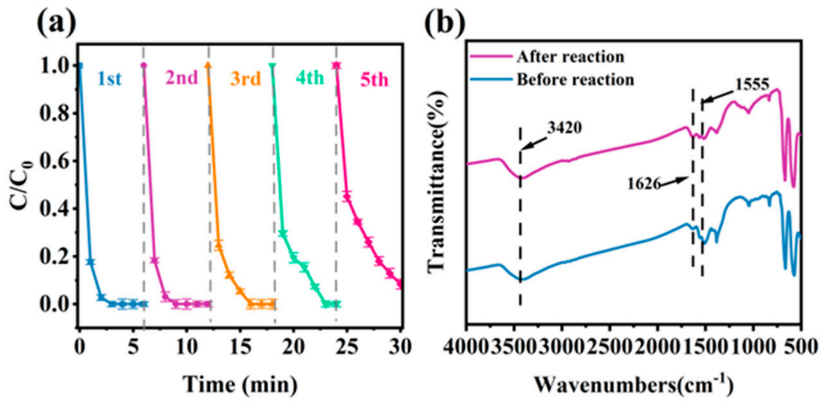

Figure S7. (a) Stability and reusability of Co_3O_4/ZnO catalyst to activate PMS for RhB Degradation. (b) FTIR spectra of Co_3O_4/ZnO before and after stability test. Experimental conditions: [RhB] = 20 mg/L, [PMS] = 0.08 mM, [catalyst] = 0.1 g/L, pH=5.7.

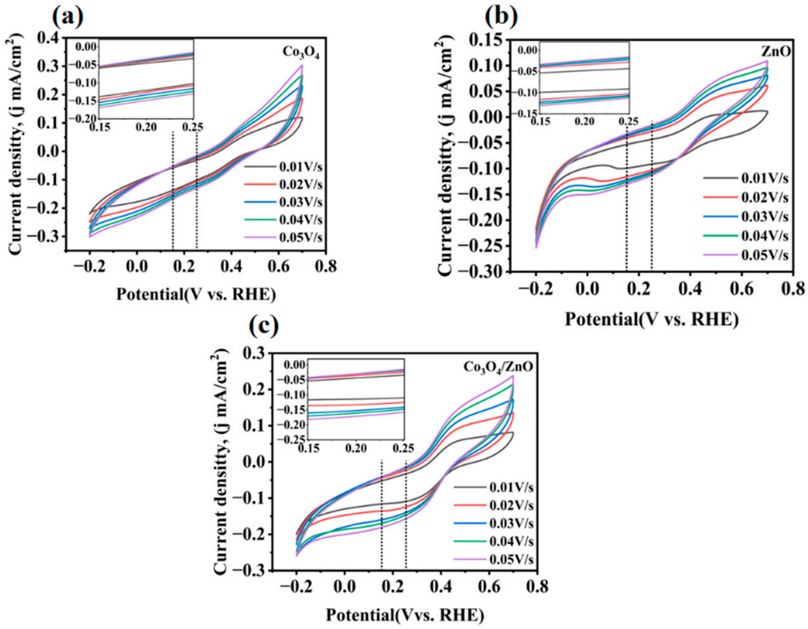

Figure S8. Cyclic voltammetry curves of (a) Co_3O_4, (b) ZnO and (c) Co_3O_4/ZnO composite at different scan rates, respectively.

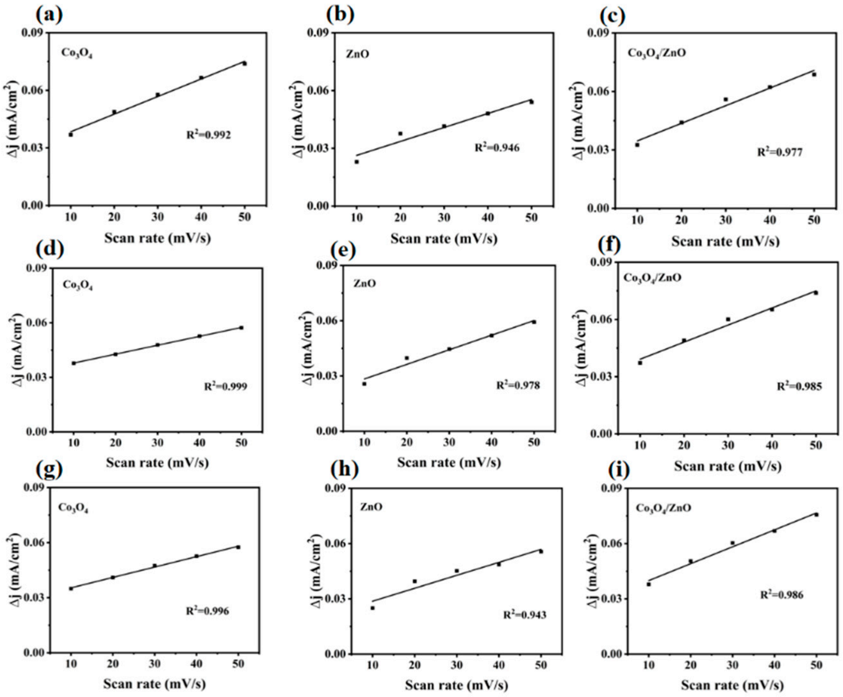

Figure S9. Plot of scan rate versus current density variation at (a-c) 0.15 V, (d-f) 0.20 V, and (g-i) 0.25 V.

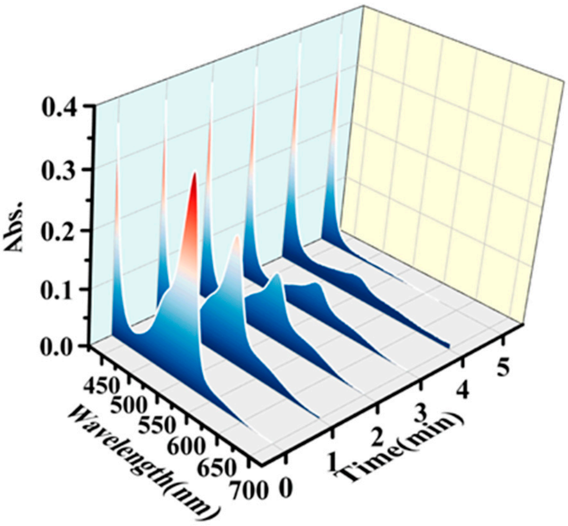

Figure S10. UV–vis absorption spectra at different times during reaction.

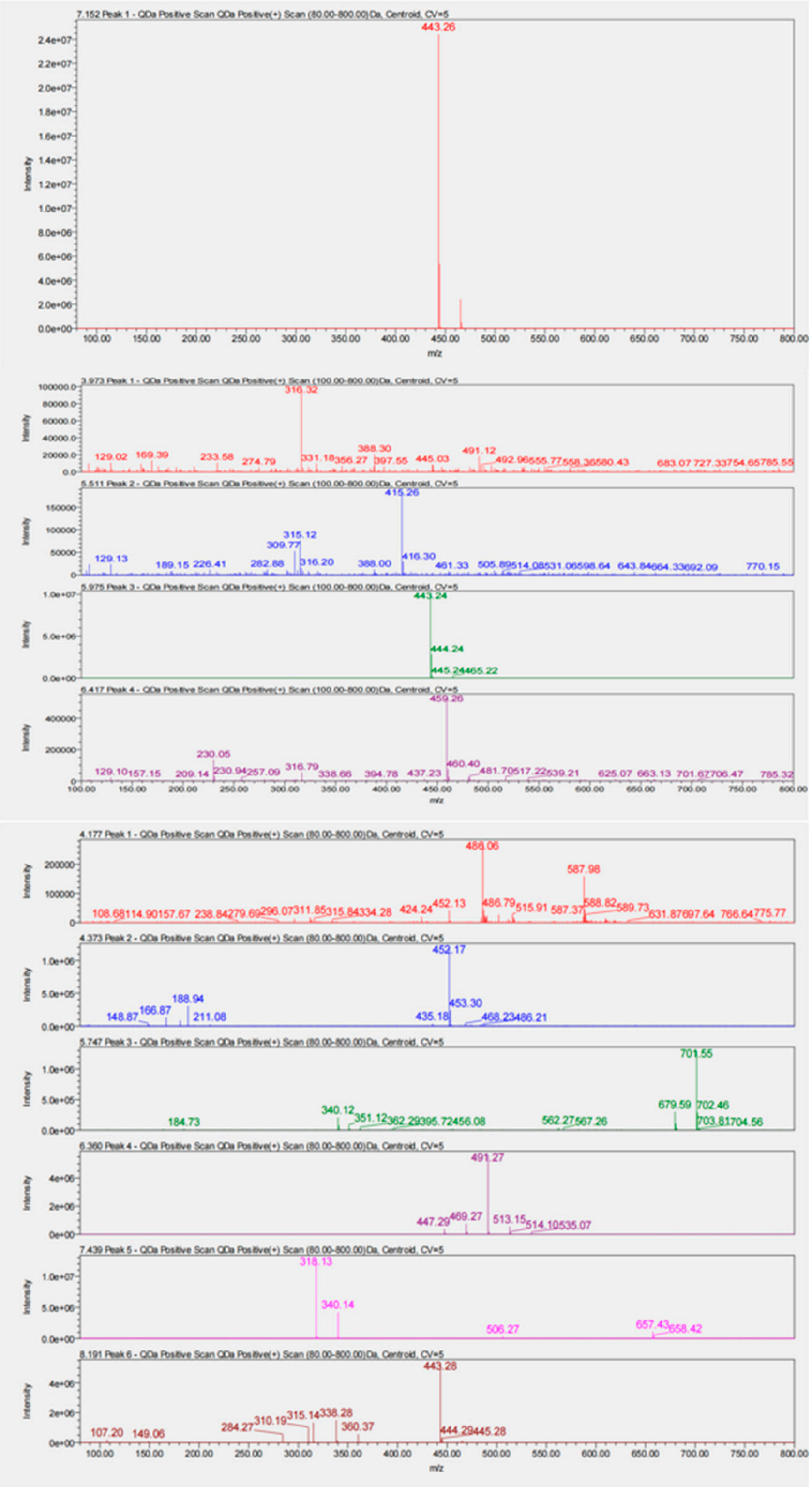
7.152 Peak 1 - QDa Positive Scan QDa Positive(+) Scan (80.00-800.00)Da, Centroid, CV=5
443.26
Intensity
m/z
3.973 Peak 1 - QDa Positive Scan QDa Positive(+) Scan (100.00-800.00)Da, Centroid, CV=5
5.511 Peak 2 - QDa Positive Scan QDa Positive(+) Scan (100.00-800.00)Da, Centroid, CV=5
5.975 Peak 3 - QDa Positive Scan QDa Positive(+) Scan (100.00-800.00)Da, Centroid, CV=5
6.417 Peak 4 - QDa Positive Scan QDa Positive(+) Scan (100.00-800.00)Da, Centroid, CV=5
4.177 Peak 1 - QDa Positive Scan QDa Positive(+) Scan (80.00-800.00)Da, Centroid, CV=5
4.373 Peak 2 - QDa Positive Scan QDa Positive(+) Scan (80.00-800.00)Da, Centroid, CV=5
5.747 Peak 3 - QDa Positive Scan QDa Positive(+) Scan (80.00-800.00)Da, Centroid, CV=5
6.360 Peak 4 - QDa Positive Scan QDa Positive(+) Scan (80.00-800.00)Da, Centroid, CV=5
7.439 Peak 5 - QDa Positive Scan QDa Positive(+) Scan (80.00-800.00)Da, Centroid, CV=5
8.191 Peak 6 - QDa Positive Scan QDa Positive(+) Scan (80.00-800.00)Da, Centroid, CV=5

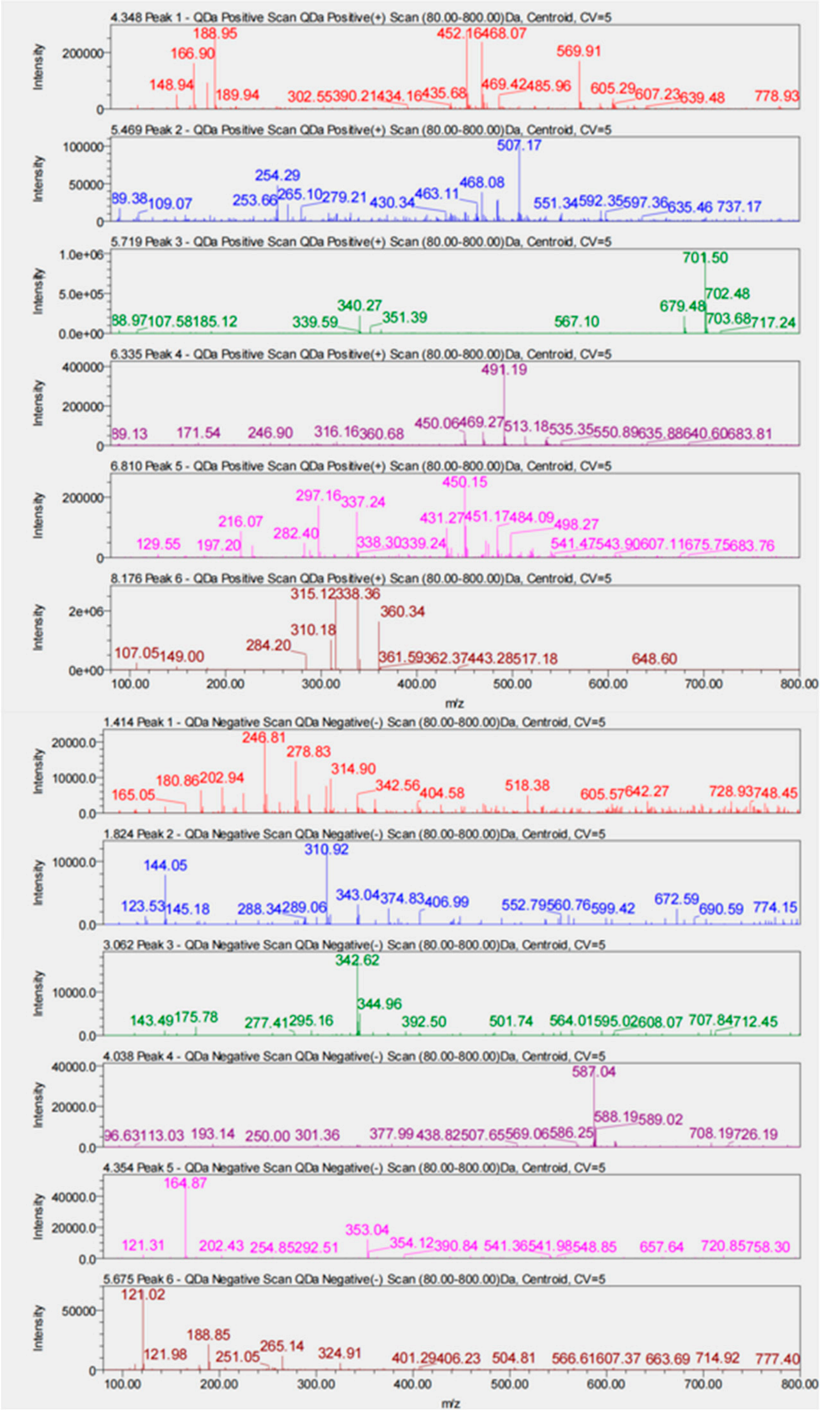

Figure S11. LC-MS chromatograms of RhB degradation intermediates at different time intervals in the Co_3O_4/ZnO/PMS system. Experimental conditions: [RhB] = 20 mg/L, [PMS] = 0.08 mM, [catalyst] = 0.1 g/L, pH=5.7.

Table S1. Comparison of the operating factors and performance of different catalyst for RhB degradation

Catalyst(g/L)	Reaction condition	Removal degree (%)	Time (min)	kobs	Ref.
g-C_3N_4/TiO_2(0.5)	[RhB] = 10 mg/L, [Catalyst] = 0.5 g/L, [PMS] = 0.1 g/L	98.5	60	-	[5]
Bi_2WO_6/BiOCl(0.2)	[RhB] = 10 mg/L, [Catalyst] = 0.1 g/L, [PMS] = 0.15 g/L	97.5	25	0.1155	[6]
$Fe_2(MoO_4)_3$-4(0.2)	[RhB] = 10 mg/L, [Catalyst] = 0.1 g/L, [PMS] = 0.2 g/L 10	98.4	30	-	[7]
DR-MoS_2/ 100(0.1)	[RhB] = 10 mg/L, [Catalyst] = 0.1 g/L, [PMS] = 0.6 g/L	92.0	25	0.0160	[8]
68 M-10/200(0.2)	[RhB] = 10 mg/L, [Catalyst] = 0.2 g/L, [PMS] = 0.06 g/L	99.0	15	-	[9]
$CoFe_2O_4$-CV(0.1)	[RhB] = 100 mg/L, [Catalyst] = 0.1 g/L, [PMS] = 0.6 g/L	99.0	15	0.4010	[10]
Co_3O_4/MoS_2@NCS	[RhB] = 30 mg/L, [Catalyst] = 0.2 g/L, [PMS] = 0.6 g/L	97.8	5	1.0370	[11]
MIL-101(Fe,Co)	[RhB] = 10 mg/L, [Catalyst] = 0.2 g/L, [PMS] = 0.4 g/L	99.0	15	0.3369	[12]
Fe-Co-Co PBA@PmPDs	[RhB] = 15 mg/L, [Catalyst] = 0.1 g/L, [PMS] = 0.4 g/L	94.3	60	0.0420	[13]

Mg/Fe_2O_3	[RhB] = 10 mg/L, [Catalyst] = 0.5 g/L, [PMS] = 0.2 g/L	96.0	120	0.0340	[14]
Co_3O_4/ZnO	[RhB] = 5 mg/L, [Catalyst] = 0.3 g/L, Light (λ=365nm)	99.7	105	-	[15]
Co_3O_4/ZnO	[RhB] = 20mg/L, [Catalyst] = 0.1 g/L, [PMS] = 0.05 g/L	100.0	6	0.8900	This work

Table S2. Comparison of different catalyst activation energies of organic pollutants.

Samples	pollutant	Ea (kJ/mol)	Reference
CuBTC-300	BPA	19.50	[16]
MIL-53-derived $CuFe_2O_4/Fe_2O_3$	SMX	43.17	[17]
ZIF-67 derived $Co_3O_4/NiCo_2O_4$	BPA	37.70	[18]
Co-based biochar	ATZ	20.60	[19]
ZIF-67-derived magnetic carbon	RhB	21.60	[20]
Co_3O_4/ZnO	RhB	2.91	This work

Table S3. Intermediates from the degradation of RhB in the Co_3O_4/ZnO/PMS system.

No.	Molecular structure	Chemical formula	m/z
RhB	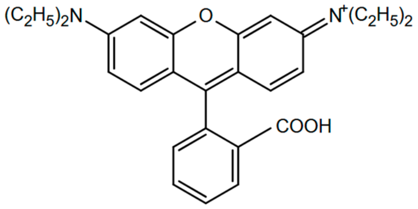	$C_{28}H_{31}O_3N_2^+$	443.26
P1	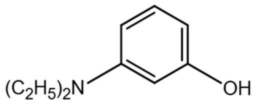	$C_{10}H_{15}NO$	164.90
P2	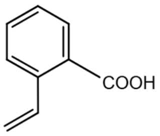	$C_9H_8O_2$	148.94
P3	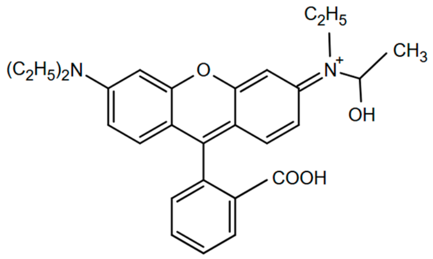	$C_{28}H_{31}N_2O_4$	459.26
P4	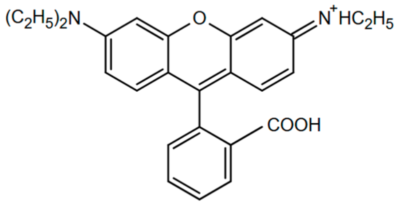	$C_{26}H_{27}O_3N_2^+$	415.26
P5	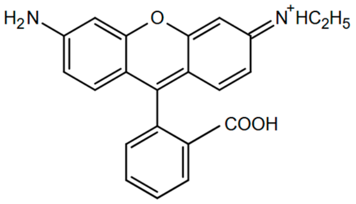	$C_{22}H_{19}O_3N_2^+$	359.64

P6	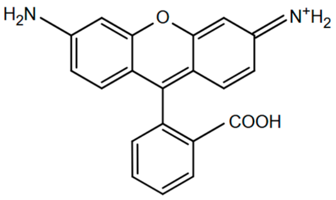	$C_{20}H_{15}O_3N_2^+$	331.85
P7	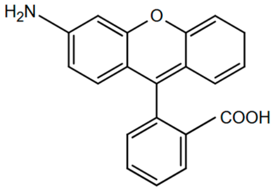	$C_{20}H_{16}NO_3$	318.13
P8	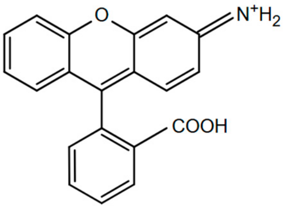	$C_{20}H_{16}O_3N^+$	316.20
P9	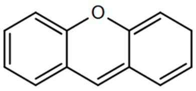	$C_{13}H_{10}O$	180.86
P10	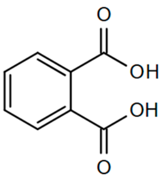	$C_8H_6O_4$	166.90
P11	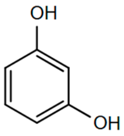	$C_6H_6O_2$	110.99
P12	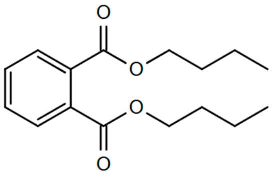	$C_{16}H_{22}O_4$	279.21
P13	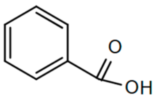	$C_7H_6O_2$	121.02

P14	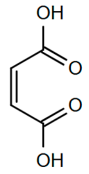	$C_4H_4O_4$	113.03
P15	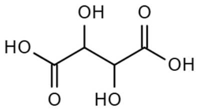	$C_4H_6O_6$	149.06
P16	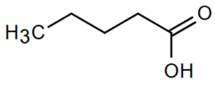	$C_5H_{10}O_2$	102.13
P17	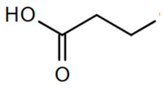	$C_4H_8O_2$	88.97

Table S4. Comparison of free radical and non-free radical pathways

comparison dimension	radical pathways	non-radical pathways
**Primary active species**	$SO4^{\cdot -}$, ·OH	$^1O_2$, High-valent metal ions (Co^{3+} and Zn^{3+})
**Detection methods**	EPR (Free radical detection) and quenching test	EPR and NBT oxidation test (detecting $^1O_2$)
**Reaction equation**	$Co^{2+} + HSO_5^- \rightarrow Co^{3+} + SO_4^{\cdot -} + OH^-$<br>$Zn^{2+} + HSO_5^- \rightarrow Zn^{3+} + SO_4^{\cdot -} + OH^-$<br>$Co^{2+} + HSO_5^- \rightarrow Co^{3+} + SO_4^{2-} + \cdot OH$<br>$Zn^{2+} + HSO_5^- \rightarrow Zn^{3+} + SO_4^{2-} + \cdot OH$<br>$SO_4^{\cdot -} + OH^- \rightarrow SO_4^{2-} + \cdot OH$<br>$SO_4^{\cdot -} + H_2O \rightarrow HSO_4^- + \cdot OH$<br>$SO_4^{\cdot -}/\cdot OH/ + RhB \rightarrow$ intermediates $\rightarrow CO_2 + H_2O$	$Co^{3+} + HSO_5^- \rightarrow Co^{2+} + SO_5^{\cdot -} + H^+$<br>$Zn^{3+} + HSO_5^- \rightarrow Zn^{2+} + SO_5^{\cdot -} + H^+$<br>$2SO_5^{\cdot -} + H_2O \rightarrow 2HSO_4^- + 1.5^1O_2$<br>$2SO_5^{\cdot -} \rightarrow S_2O_8^{2-} + ^1O_2$<br>$2SO_5^{\cdot -} \rightarrow 2SO_4^{2-} + ^1O_2HSO_5^- + SO_5^{2-} \rightarrow SO_4^{2-} + HSO_4^- + ^1O_2$<br>$Olattice + HSO_5^- \rightarrow HSO_4^-$<br>$Co^{3+} + e^- \rightarrow Co^{2+}$<br>$^1O_2 + RhB \rightarrow$ intermediates $\rightarrow CO_2 + H_2O$

Reference

1. Li F, Wei J, Wang D, Han Y, Han D, Gong J. Ce-doped $CuCoO_2$ delafossite with switchable PMS activation pathway for tetracycline degradation. *Chem. Eng. J.* **2024**, 481, 148633. https://doi.org/10.1016/j.cej.2024.148633

2. Zhu H, Guo A, Xian L, Wang Y, Long Y, Fan G. Facile fabrication of surface vulcanized Co-Fe spinel oxide nanoparticles toward efficient 4-nitrophenol destruction. *J. Hazard. Mater.* **2022**, 430, 128433. https://doi.org/10.1016/j.jhazmat.2022.128433

3. Li D, Zhang G, Li W, Fang Z, Liu H, Lv W, Liu G. Magnetic nitrogen-doped carbon nanotubes as activators of peroxymonosulfate and their application in non-radical degradation of sulfonamide antibiotics. *J. Clean Prod.* **2022**, 380, 135064. https://doi.org/10.1016/j.jclepro.2022.135064

4. Chang L, Xue X, Deng Q, Xie X, Zhang X, Cheng C, Chai H, Huang Y. Modulating the electronic structure of Co center via MgO@C co-doping for PMS activation to remove levofloxacin. *Sep. Purif. Technol.* **2023**, 321, 124151. https://doi.org/10.1016/j.seppur.2023.124151

5. Zhang B, Wu M, Chen Z, Dong L, Li B, Tao L, Wang H, Li D. Fabrication of novel direct Z-scheme + isotype heterojunction photocatalyst g-C_3N_4/TiO_2 with peroxymonosulfate (PMS) activation synergy and 2D/0D structure. *Catal. Sci. Technol.* **2022**, 12, 7199-7207. https://doi.org/10.1039/d2cy01387h

6. Xu M, Lu M, Yang Y, Ai L, Fan H, Guo N, Wang L. Efficient degradation of pollutants by Bi_2WO_6/BiOCl heterojunction activated peroxymonosulfate: Performance and mechanism. *J. Environ. Chem. Eng.* **2024**, 12, 112156. https://doi.org/10.1016/j.jece.2024.112156

7. Chang L, Xie X, Zhang X, Chai H, Huang Y. Overlooked key role of Mo(VI) in $Fe_2(MoO_4)_3$ for peroxymonosulfate activation with $^1O_2$ dominated degradation pathway. *Sep. Purif. Technol.* **2023**, 322, 124360. https://doi.org/10.1016/j.seppur.2023.124360

8. Li S, Ning X, Hao P, Cao Y, Xie J, Hu J, Lu Z, Hao A. Defect-rich MoS_2 piezocatalyst: Efficient boosting piezocatalytic activation of PMS activity towards degradation organic pollutant. *Dyes Pigm.* **2022**, 206, 110678. https://doi.org/10.1016/j.dyepig.2022.110678

9. Chen X, Peng C, Luo F, Du G, Zhang Y, Zhao J, Jiang L, Su H, Shan S, Hu T. Novel MIL-68(Fe)/MoS_2 composites promote peroxymonosulfate activation for efficiently removing Rhodamine B. *J. Alloys Compd.* **2024**, 976, 173369. https://doi.org/10.1016/j.jallcom.2023.173369

10. Jin Z, Zhao X, Zhang M, Li Y, Guo J, Lan Y, Chen C. Waste self-heating bag derived $CoFe_2O_4$ composite enhances peroxymonosulfate activation: Performance, mechanism, and adaptability under high-salinity conditions. *J. Water Process. Eng.* **2024**, 60, 105221. https://doi.org/10.1016/j.jwpe.2024.105221

11. Jiang R, Zhong D, Xu Y, Chang H, He Y, Zhang J, Liao P. Chitosan derived N-doped carbon anchored Co_3O_4-doped MoS_2 nanosheets as an efficient peroxymonosulfate activator for degradation of dyes. *Int. J. Biol. Macromol.* **2024**, 265, 130519. https://doi.org/10.1016/j.ijbiomac.2024.130519

12. Xiao Z, Wu R, Shu T, Wang Y, Li L. Synthesis of Co-doped Fe metal–organic framework MIL-101(Fe,Co) and efficient degradation of organic dyes in water. *Sep. Purif. Technol.* **2023**, 304, 122300. https://doi.org/10.1016/j.seppur.2022.122300

13. Zeng L, Xiao L, Shi X, Wei M, Cao J, Long Y. Core-shell Prussian blue analogues@ poly(m-phenylenediamine) as efficient peroxymonosulfate activators for degradation of Rhodamine B with reduced metal leaching. *J. Colloid Interface Sci.* **2019**, 534, 586-594. https://doi.org/10.1016/j.jcis.2018.09.074

14. Guo S, Liu M, You L, Cheng G, Li J, Zhou K. Oxygen vacancy induced peroxymonosulfate

activation by Mg-doped Fe_2O_3 composites for advanced oxidation of organic pollutants. *Chemosphere* **2021**, 279, 130482. https://doi.org/10.1016/j.chemosphere.2021.130482

15. Mohamed Reda G, Fan H, Tian H. Room-temperature solid state synthesis of Co_3O_4/ZnO p–n heterostructure and its photocatalytic activity. *Adv. Powder Technol.* **2017**, 28, 953-963. https://doi.org/10.1016/j.apt.2016.12.025

16. Wang M-M, Cui Y-K, Wen J-T, Wang Y-S, Jia M-H, He S-Z, Wang W-K, Xu J. Flexible regulation of persulfate activation mechanisms through tuning Cu valence in CuBTC-derived copper oxide catalysts for improved pollutant degradation. *Chem. Eng. J.* **2023**, 476, 146565. https://doi.org/10.1016/j.cej.2023.146565

17. Asif A H, Rafique N, Hirani R A K, Shi L, Wang Y, Duan X, Yin Y, Sun H. MIL-53(Fe) derived magnetic $CuFe_2O_4$/Fe_2O_3 composite for catalytic oxidation of sulfamethoxazole via peroxymonsulfate activation. *Chem. Eng. J.* **2023**, 469, 143915. https://doi.org/10.1016/j.cej.2023.143915

18. Wang M, Cui Y, Cao H, Wei P, Chen C, Li X, Xu J, Sheng G. Activating peroxydisulfate with Co_3O_4/$NiCo_2O_4$ double-shelled nanocages to selectively degrade bisphenol A – A nonradical oxidation process. *Appl. Catal. B-Environ.* **2021**, 282, 119585. https://doi.org/10.1016/j.apcatb.2020.119585

19. Liu B, Guo W, Wang H, Si Q, Zhao Q, Luo H, Ren N. Activation of peroxymonosulfate by cobalt-impregnated biochar for atrazine degradation: The pivotal roles of persistent free radicals and ecotoxicity assessment. *J. Hazard. Mater.* **2020**, 398, 122768. https://doi.org/10.1016/j.jhazmat.2020.122768

20. Xiao L, Xu S, Zhang Y, Song Y, Wu D, Jiang K. Cellulose nanocrystals mediated spontaneous weaving of ZIF-67 hybrid networks: Enhanced peroxymonosulfate activation and rapid removal of dye. *Surf. Interfaces* **2024**, 50, 104539. https://doi.org/10.1016/j.surfin.2024.104539
